# Correction to “Exploring the Landscape of Extracellular Vesicle Application for Skin and Plastic Surgery: A Bibliometric Analysis From 2003 to 2023”

**DOI:** 10.1111/srt.70169

**Published:** 2025-04-10

Q. Liu, H. Zhang, Y. Zhu, L. Jia, R. Guo, Y. Sun, and J. Xu, “Exploring the Landscape of Extracellular Vesicle Application for Skin and Plastic Surgery: A Bibliometric Analysis From 2003 to 2023,” *Skin Research and Technology* 30, no. 8 (2024): e13879, https://doi.org/10.1111/srt.13879.

In the published article, to reported query for the search strategy in the Web of Science Core Collection (WoSCC) is partially incorrect. The correct query is presented below (changes in bold):

(TS = (stem cell*) OR TS = (SC) OR TS = (SCs) OR TS = (MSC*) OR TS = (Mesenchymal stem cell*)) AND (TS = (“extracellular vesicle*” OR “EV” OR “EVs” OR “exosome*” OR “microvesicle*” OR “shed microvesicle*” OR “small microvesicle* ”OR “sMV*”)) AND (TS = (skin) OR TS = (Dermatology) OR TS = (Dermatologic Surgical Procedure*) OR TS = (Procedure*, Dermatologic Surgical) OR TS = (Surgical Procedure*,Dermatologic) OR TS = (Cutaneous* Surgical Procedures) OR TS = (Procedure*,Cutaneous Surgical) OR TS = (Surgical Procedure*,Cutaneous) OR TS = (Skin Surger*) OR TS = (Surger*,Skin) OR TS = (Cutaneous Surger*) OR TS = (Surger*,Cutaneous) OR TS = (Dermatologic Surger*) OR TS = (Surger*,Dermatologic) OR TS = (Surger*, Plastic) OR TS = (Plastic Surger*) OR TS = (Esthetic Surger*) OR TS = (Surger*, Esthetic) OR TS = (Surger*,Cosmetic) OR TS = (Cosmetic Surger*) OR TS = (Wound Healing*) OR TS = (Healing*, Wound) OR TS = (Re‐Epithelialization) OR TS = (Wound Epithelialization) OR TS = (Epithelialization, Wound)) AND (DOP = (2003‐01‐01/2023‐12‐31))

The authors apologize for this error and for any inconvenience it may have caused.

